# Effects of NMES Combined with Water-Based Resistance Training on Muscle Coordination in Freestyle Kick Movement

**DOI:** 10.3390/s26020673

**Published:** 2026-01-20

**Authors:** Yaohao Guo, Tingyan Gao, Jun Liu

**Affiliations:** 1 School of Mathematical Sciences, Fudan University, Shanghai 200433, China; guoyaohao_fdu@163.com; 2 Faculty of Physical Education, Fudan University, Shanghai 200433, China; gaotingyan@fudan.edu.cn

**Keywords:** neuromuscular electrical stimulation, aquatic strength training, muscle synergy analysis, lower limb biomechanics, National Level swimming, motor control adaptation, underwater electromyography, sports performance enhancement

## Abstract

**Background:** This study aimed to explore the effects of neuromuscular electrical stimulation (NMES) combined with water-based resistance training on muscle activation and coordination during freestyle kicking. **Methods:** Thirty National Level male freestyle swimmers were randomly assigned to an experimental group (NMES + water-based training) or a control group (water-based training only) for a 12-week intervention. The experimental group received NMES pretreatment before each session. Underwater surface electromyography (sEMG) synchronized with high-speed video was used to collect muscle activation data and corresponding kinematic information during the freestyle kick. The sEMG signals were then processed using time-domain analysis, including integrated electromyography (iEMG), which reflects the cumulative electrical activity of muscles, and root mean square amplitude (RMS), which indicates the intensity of muscle activation. Non-negative matrix factorization (NMF) was further applied to extract and characterize muscle synergy patterns. **Results:** The experimental group showed significantly higher iEMG and RMS values in key muscles during both kicking phases. Within the core propulsion synergy, muscle weighting of vastus medialis and biceps femoris increased significantly, while activation duration of the postural adjustment synergy was shortened. The number of synergies showed no significant difference. **Conclusions:** NMES combined with water-based resistance training enhances muscle activation and optimizes neuromuscular coordination strategies, offering a novel approach to improving sport-specific performance.

## 1. Introduction

Neuromuscular Electrical Stimulation (NMES) is a non-invasive physical therapy and training technique that induces muscle tetanic contractions independent of central nervous system commands by stimulating motor nerves with external electrical currents [1]. In recent years, NMES applications have expanded beyond traditional rehabilitation medicine into athletic training and performance enhancement in competitive sports. Research indicates that NMES effectively recruits high-threshold Type II muscle fibers, improves intermuscular coordination, and assists in maintaining and rebuilding movement patterns during fatigue or injury [2]. It has become a valuable supplement to strength and power training systems in sports such as soccer, track and field, and basketball [3,4]. Consequently, exploring novel training models that integrate NMES with various sport-specific training methods represents a significant current research direction in the science of athletic training.

In motor control science, muscle synergy theory provides a crucial framework for understanding how the central nervous system efficiently coordinates multiple muscles to execute complex movements [5]. This theory posits that the CNS does not independently control each muscle but simplifies motor control by invoking a limited set of pre-programmed synergistic modules (Muscle Synergies) with specific weighting combinations and temporal activation patterns [6]. For complex, cyclic, multi-joint coordinated movements like swimming, analyzing muscle synergy patterns is crucial for revealing action efficiency, technique optimization, and neural adaptation mechanisms. The fluidity and propulsive force of the freestyle kick fundamentally depend on whether relevant muscle groups can form efficient, stable synergistic patterns [7]. Therefore, investigating the effects of training interventions on muscle synergy structures is a key pathway to deepen understanding of the neuromuscular adaptation mechanisms underlying performance enhancement.

As a full-body, cyclical competitive sport, swimming performance is highly dependent on optimizing technical efficiency and energy output [8]. Freestyle swimming, due to its typical technical structure, is often used as a key model in swimming biomechanics research [9]. Although the kick contributes only about 10–15% of total propulsion [10,11], it plays an irreplaceable role in maintaining a streamlined body posture, balancing the upper-body stroke, and coordinating the rhythmic movement of the entire body [12]. Research indicates that efficient freestyle kicking relies on a hip-dominant “whiplash” mechanism, involving precise sequential activation and coordinated work of multiple muscle groups—including the gluteus maximus, quadriceps, hamstrings, and triceps sure—through the kinetic chain [12]. Poor kicking technique not only increases energy expenditure but may also directly impair swimming speed due to compromised body posture and increased resistance [13]. Therefore, elucidating the muscle activation patterns and neural coordination mechanisms underlying freestyle kicking holds clear practical significance for enhancing athletes’ competitive performance.

Currently, strength training for swimmers is predominantly conducted on land. While this aids in developing foundational muscle strength, it often lacks alignment with the specific movement patterns and force generation environment (e.g., fluid resistance, lack of fixed support) encountered in the water [14]. Water-based resistance training can partially simulate the technical environment of swimming, effectively enhancing kicking power and endurance [15]. However, effective intervention methods remain lacking for further enhancing the strong neuromuscular effects of such training, particularly in optimizing the central nervous system’s coordinated control strategies for sport-specific movements.

With advancements in sports biomechanics and training monitoring technologies, motion analysis systems based on wearable sensors (e.g., surface electromyography sensors) have been widely applied in swimming technique diagnosis and training evaluation [16]. These sensors enable real-time, synchronous collection of multi-channel electromyography and kinematic data in aquatic environments, providing technical support for in-depth quantitative analysis of muscle activation patterns and temporal coordination [17]. Integrating sensor technology with NMES and aquatic resistance training creates a dual intervention: NMES directly enhances peripheral neural drive and muscle recruitment capacity, while aquatic resistance training facilitates biomechanically compliant sport-specific movement patterns under central nervous system control [18]. Whether this combination produces a synergistic enhancement effect—not only boosting muscle output capacity but also optimizing intermuscular coordination patterns to improve overall kick efficiency—remains a scientific question worthy of in-depth exploration.

Given this, the present study aims to utilize underwater sEMG technology to investigate the effects of a 12-week NMES combined with aquatic resistance training (primarily performed with fins) on muscle activation levels and coordination patterns during the kicking motion of National Level freestyle swimmers. By establishing a control group undergoing water resistance training alone, this study aims to reveal the role of NMES in enhancing sport-specific training effects and optimizing neuromuscular control strategies, thereby providing new theoretical foundations and practical solutions for scientific swimming training.

## 2. Participants and Methods

### 2.1. Participants

The sample size for this study was determined a priori using G*Power software (version 3.1.9.7; Heinrich Heine University Düsseldorf, Düsseldorf, Germany) [19]. For a repeated measures analysis of variance (ANOVA), a medium effect size (f = 0.25), an alpha level of 0.05, and a statistical power (1 − β) of 0.80 were specified. The calculation indicated a minimum required sample size of 26 participants. To account for potential dropouts, 30 National Level male freestyle swimmers were recruited, all meeting the following performance-based inclusion criteria.

Participants were classified as “Highly-Trained/National Level” according to the framework proposed by McKay et al. (2022) [20]. This classification corresponds to athletes who compete at the national level, engage in systematic high-volume training (≥9 sessions per week), and have achieved a top-three finish at the Chinese National Swimming Championships. All participants were specialists in freestyle events, holding a National Class 1 Athlete certification, and had achieved a podium finish (top three) at the Chinese National Swimming Championships (long course). Their mean (±SD) age, height, body mass, and training history were 22.8 ± 3.2 years, 181.5 ± 5.1 cm, 72.3 ± 4.7 kg, and 10.5 ± 2.8 years, respectively.

Inclusion criteria were: male sex; age between 18 and 30 years; possession of National Class 1 Athlete status or higher; a top-three finish in a national-level freestyle competition; physical capability to participate in aquatic training and NMES intervention; and voluntary participation with written informed consent.

Exclusion criteria comprised: a history of acute lower limb or lumbar injury or surgery within the preceding six months; diagnosed neurological disorders, skin allergies, or lesions at planned electrode sites; a history of severe cardiovascular disease, epilepsy, or other contraindications to electrical stimulation; recent use of medications known to affect neuromuscular function; or any condition preventing the completion of the testing and training protocols.

This study received approval from the institutional ethics committee and was conducted in strict accordance with the ethical principles of the Declaration of Helsinki.

### 2.2. Experimental Equipment

(1) Mini Wave infinity Water Proof

**Figure 1:**
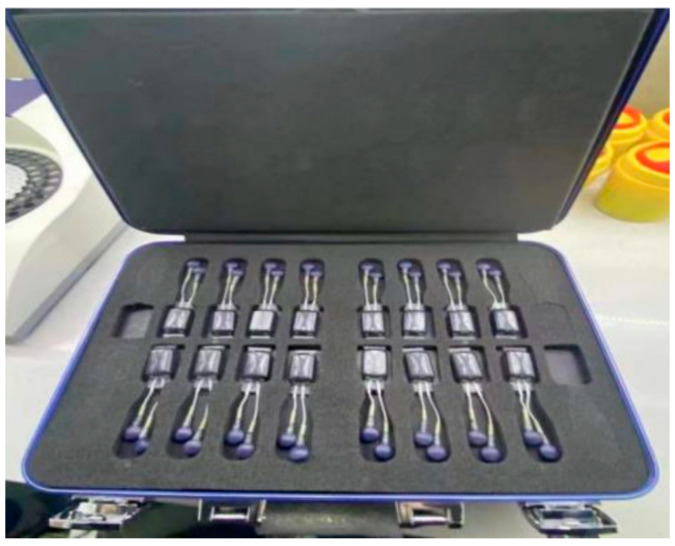
Underwater Surface Electromyography Sensor.

A wireless underwater sEMG system (Mini Wave Infinity, Cometa Systems, Milan, Italy) was used to acquire muscle activity data. This system is specifically designed for aquatic applications, featuring an IP68 waterproof rating for the sensors and transmitter. The sEMG signals were recorded at a sampling frequency of 2000 Hz. Sensor placement and fixation procedures to ensure signal reliability are detailed in Section 2.6 (Figure 1).

(2) Compex SP 8.0 Electrical Stimulator

**Figure 2:**
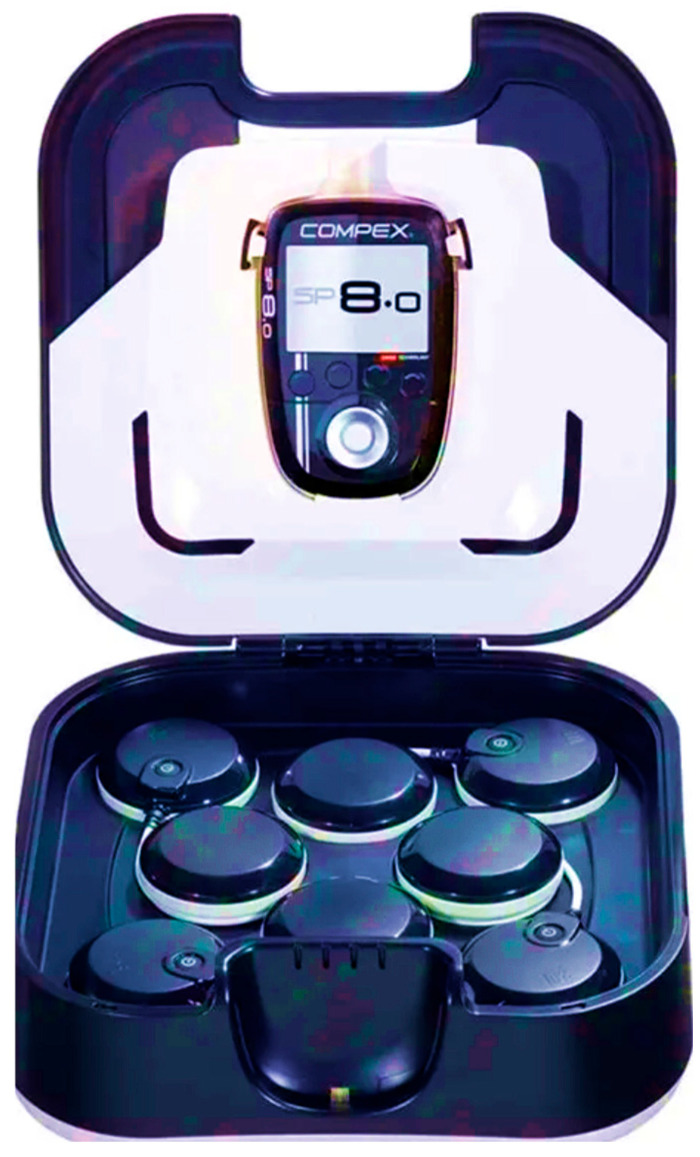
Compex SP 8.0 Electrical Stimulator.

The neuromuscular electrical stimulation (NMES) intervention was delivered using a portable electrical stimulator (Compex SP 8.0, DJO Global, Guildford, UK). The device was programmed to deliver a biphasic, symmetrical rectangular waveform. For the pretreatment protocol, stimulation parameters were set at a frequency of 50 Hz and a pulse width of 200 µs. Self-adhesive, reusable stimulation electrodes (Compex, 50 × 50 mm) were used. During each session, the electrodes were firmly positioned over the muscle bellies of the target muscles (i.e., vastus medialis, vastus lateralis, and biceps femoris) according to standard anatomical landmarks. Stimulation intensity was individually adjusted to a level corresponding to 80–90% of the participant’s maximum tolerated contraction, ensuring visible muscle contraction without discomfort (Figure 2).

(3) Underwater High-Speed Camera

An underwater high-speed digital camera (GoPro HERO10 Black, GoPro Inc., San Mateo, CA, USA), mounted on a fixed tripod and housed in a waterproof casing, was used for kinematic recording. The camera was positioned perpendicular to the plane of motion at a distance of 5 m. Video data were captured at a frame rate of 200 Hz with a resolution of 1920 × 1080 pixels, synchronized with the sEMG system via an external trigger for temporal alignment.

(4) Method Description

Standard disposable Ag/AgCl surface electrodes (Ambu BlueSensor N, Ambu A/S, Ballerup, Denmark) were used for sEMG recording. Adhesive medical tape (3M Tegaderm, 3M, Saint Paul, MN, USA) and waterproof adhesive film were used to secure and waterproof the electrodes. Standard skin preparation materials (isopropyl alcohol, cotton swabs, disposable razors) and a digital stopwatch were also employed during testing procedures.

### 2.3. Test Movements

(1) Maximum Voluntary Contraction (MVC) Test: Prior to the main experimental trials, each of the ten target muscles (as specified in Section 2.5) underwent an MVC test for electromyographic (EMG) signal normalization. For each muscle, participants performed three maximal isometric contractions against manual resistance, each lasting approximately 5 s, with a 60 s rest interval between contractions. The trial with the highest root mean square (RMS) amplitude over a stable 1 s window was selected, and its value was used to normalize the dynamic EMG signals collected during the subsequent freestyle kick test.
(2) Freestyle Kick Performance Test: The dynamic performance test consisted of a 25 m maximal freestyle kick sprint in a short-course pool (25 m length). Participants initiated the trial by pushing off the wall in a streamlined position, using only a freestyle (flutter) kick for propulsion while holding a kickboard with their arms extended. They were instructed to maintain maximal kicking effort throughout the entire distance. Each participant performed three trials, with a standardized 5 min passive rest interval between trials to minimize fatigue. During each trial, synchronized sEMG and high-speed video data were continuously collected. For data analysis, one complete and representative kick cycle (from the highest point of one leg’s upward swing to its return to the same position) was extracted from the middle 15 m segment of each participant’s fastest trial. This approach ensured the analysis of data from a consistent, high-intensity, and non-fatigued phase of the movement.

### 2.4. Intervention Methods

Following baseline testing, participants were randomly allocated into one of two groups (n = 15 per group) using a computer-generated randomization sequence. There were no significant differences between groups at baseline regarding age, height, body mass, training history, or initial kicking performance (all *p* > 0.05). The experimental group (EXP) received a combined intervention of neuromuscular electrical stimulation (NMES) pretreatment followed by aquatic resistance training (ART), while the control group (CON) performed ART only. Both groups undertook a 12-week training program comprising three supervised sessions per week (≥48 h between sessions). Each 60 min session followed a standardized structure: a 10 min warm-up (dynamic stretching, joint mobilization, 400 m low-intensity swimming), 45 min of main intervention, and a 5 min cool-down (400 m relaxed swimming, static stretching).

The specific interventions for each group are summarized in Table 1. All ART sessions were conducted using short-bladed fins (Finis Short Blade, Finis, Inc., Livermore, CA, USA) and followed the principle of progressive overload. Load was re-evaluated every two weeks; if an athlete could complete 12 repetitions per set with proper technique, the load was increased by 5–10% for the following training block.

All training sessions were supervised by experienced swimming coaches and researchers. NMES application was administered by trained personnel to ensure consistency, safety, and adherence to the prescribed parameters.

### 2.5. Data Acquisition

Based on the principles of underwater kicking techniques, the characteristics of lower-body swimming training, combined with knowledge of exercise anatomy, and drawing upon previous research findings [21], a total of 10 muscles were selected for analysis: Tibialis anterior, Gastrocnemius medialis, Gastrocnemius lateralis, Adductor longus, Adductor magnus, Biceps femoris, Gluteus maximus, Rectus abdominis, Latissimus dorsi, Trapezius. EMG sensors were applied in the above sequence, all positioned on the subject’s right side.

Subjects first underwent skin preparation: removing surface hair and cleaning the skin with alcohol. Surface electrode positions were then determined using SENIAM guidelines [22]. Disposable Ag/AgCl gel electrodes were placed parallel to the muscle fiber orientation at the most prominent point of the muscle belly, maintaining a consistent 2 cm distance between electrodes. The reference electrode is placed on an adjacent area unaffected by the test muscle’s fibers, such as the tibia. Ensure the placement of the EMG electrodes does not interfere with normal movement. Secure the reference electrode with medical adhesive tape and skin film to prevent loosening or detachment during movement.

**Figure 3:**
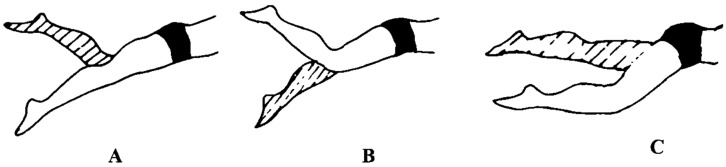
Phases of the Freestyle Kick: (**A**) is the upward swing phase, (**B**) is the downward kick phase, (**C**) is the transition phase.

This study employed synchronized data acquisition using the Cometa wireless underwater surface electromyography system and a 200 Hz underwater high-speed camera to record electromyographic signals and corresponding kinematic data from ten target muscles during the freestyle kick phase. Electrode placement strictly adhered to SENIAM standards. Based on the biomechanical characteristics of the kick, each kick cycle (i.e., one complete movement by the left and right legs) was divided into three consecutive phases: the upward swing phase (thigh driving the lower leg upward to the highest point), the downward kick phase (lower leg accelerating downward to the lowest point), and the transition phase (the transition process from the lowest point back upward) (see Figure 3 for schematic). The acquired electromyographic signals will subsequently undergo non-negative matrix factorization (NMF) to extract muscle coordination characteristics.

### 2.6. Data Processing

#### 2.6.1. Data Extraction and Preprocessing

All signal processing, time-domain analysis (iEMG, RMS), and muscle synergy extraction via NMF were performed using custom scripts written in MATLAB (R2023a, The MathWorks Inc., Natick, MA, USA).

To address the challenges of sensor displacement and signal distortion in underwater conditions, we implemented a multi-layered fixation protocol for all sEMG electrodes. Prior to sensor placement, the skin was shaved, abraded, and cleaned with alcohol to ensure optimal adhesion. Each disposable Ag/AgCl electrode was secured parallel to the muscle fiber direction using waterproof medical adhesive tape (3M Tegaderm™, United States of America) and further reinforced with a waterproof elastic mesh sleeve (Aqua Cover, Cometa Systems 5.1). The reference electrode was positioned on the ipsilateral tibial tuberosity, a stable and minimally moving anatomical landmark. To verify signal integrity throughout the test, we visually inspected electrode positioning before and after each trial and monitored raw EMG signals in real-time using Cometa’s proprietary software. Trials with noticeable signal dropout or movement artifacts (e.g., sudden amplitude shifts inconsistent with muscle activation) were excluded from analysis. Additionally, the 20–400 Hz band-pass filter applied during post-processing further attenuated low-frequency motion artifacts and high-frequency noise. This protocol ensured reliable sEMG acquisition across all ten monitored muscles during high-frequency kicking movements.

The data acquired synchronously from the wireless underwater sEMG system (sampling at 2000 Hz) and the fixed underwater high-speed camera (recording at 200 Hz) were analyzed. The camera was mounted on a fixed tripod, positioned perpendicular to the swimming lane at the midpoint (12.5 m) to capture the sagittal plane motion. Synchronization between the sEMG and video systems was achieved via an external hardware trigger that sent a simultaneous start pulse to both devices at the beginning of each trial. Each complete kick cycle was defined based on kinematic characteristics (from the highest point of the upward swing of one leg to its return to the same upward swing point) [23,24]. The sEMG data corresponding to each cycle were extracted using the synchronized timestamps for subsequent analysis. The raw EMG signals were first processed with a 4th-order Butterworth band-pass filter (20–400 Hz) to remove movement artifacts and high-frequency noise. Subsequently, the signals were full-wave rectified and smoothed using a 4th-order low-pass Butterworth filter with a cutoff frequency of 20 Hz to obtain the linear envelope. All EMG data were then normalized to the peak value obtained during the maximum voluntary isometric contraction (MVC) test for each corresponding muscle. Finally, the time axis of each kick cycle was normalized to 100 data points using linear interpolation to account for variations in cycle duration [25].

#### 2.6.2. Electromyographic Time-Domain Analysis

iEMG (Integrated Electromyogram) is a quantitative metric derived by time-integrating rectified surface electromyography signals, reflecting the cumulative total of muscle electrical activity over a specific time period [26]. This metric comprehensively represents the overall mobilization level of the neuromuscular system, with its value influenced by the number of recruited motor units, discharge frequency, and muscle contraction duration. The calculation formula is as follows:

$$iEMG\;=\sum_{n=1}^{N}\left|x_{n}\right|$$

RMS (root mean square amplitude) is obtained by calculating the average square root of the signal’s squared values, with its physical significance equivalent to the signal’s standard deviation [27]. RMS places greater emphasis on reflecting the contribution of high-amplitude signal components, and is therefore closely related to the degree of synchronized discharge in motor units and the recruitment intensity of fast-twitch muscle fibers. The calculation formula is as follows:

$$RMS=\sqrt{\int_{t}^{t+T}{EMG}^{2}\left(t\right)dt/T}$$

#### 2.6.3. Muscle Synergy Extraction

The NMF algorithm is employed to extract muscle synergy features from electromyography data [28]. The muscle activity matrix D(t) is decomposed into time-invariant synergy vectors Wi (muscle weights) and time-varying activation coefficients Ci(t), with the reconstruction formula defined as:

$$D(t)=\sum_{i=1}^{N_{syn}}\;C_{i}(t)W_{i}$$

Among them N_syn_ Indicates the number of synergies.

To determine the optimal number of synergies, synergies ranging from 1 to 14 were iteratively extracted. The criterion for selection was the minimum number of synergies required to explain over 90% of the variance in electromyographic reconstruction (VAF). The VAF is calculated using the following formula:

$$\mathrm{VAF}\;=\;1\;-\;\frac{\mathrm{SSE}}{\mathrm{SST}}$$

SST denotes the sum of squares of total, while SSE represents the sum of squares of error. The NMF iteration is initialized between 0 and the maximum electromyographic value. Iteration ceases when VAF exceeds 90%, and the solution with the highest VAF is selected for subsequent analysis.

#### 2.6.4. Muscle Synergy Clustering and Matching

K-means clustering (using squared Euclidean distance as the metric, repeated 1000 times) was employed to identify representative synergy vectors for each group. The optimal number of clusters was determined using the Gap statistic. When Gap(k) ≥ Gap(k + 1) − sd(k + 1) (where sd(k) is the standard deviation of the reference dataset), the k value was selected as the number of clusters. Following clustering, synergistic patterns from other groups were assigned to corresponding reference synergies based on muscle weight correlation coefficients (threshold 0.6) to enable intergroup synergistic pattern comparison analysis. The analysis method employs K-means clustering combined with the Gap statistic to determine the optimal number of clusters, followed by synergistic pattern matching using Pearson correlation coefficients (threshold ≥ 0.6). The software used was MATLAB (R2023a, The MathWorks Inc., Natick, MA, USA).

#### 2.6.5. Statistical Analysis

All experimental data underwent statistical processing and analysis using Excel 2010 (Microsoft Corporation, Redmond, WA, USA) and SPSS 26.0 (IBM Corporation, Armonk, NY, USA). A 2 × 2 (group: experimental vs. control × time: pre- vs. post-intervention) repeated measures ANOVA was performed to analyze the differences in IEMG, RMS, number of synergies, muscle weight, activation duration, peak activation time, and onset time between the two groups. In case of a significant main or interaction effect, Bonferroni-adjusted post hoc pairwise comparisons were conducted. Partial eta squared (ηp^2^) was computed as a measure of effect size for significant ANOVA results, with ηp^2^ ≥ 0.01, ≥0.06, and ≥0.14 interpreted as small, medium, and large effects, respectively. The significance level was set at α = 0.05.

## 3. Results

### 3.1. Effects of NMES Combined with Resistance Training on Muscle Activation During the Kick Phase in Freestyle Swimmers

As shown in Table 2, significant time × group interaction effects were observed for iEMG data in the gastrocnemius (*p* = 0.021), vastus medialis (*p* = 0.011), vastus lateralis (*p* = 0.010), and gluteus maximus (*p* = 0.035) muscles during the upward swing phase. Simple effects analysis revealed no significant differences in iEMG between the experimental and control groups for the gastrocnemius, vastus medialis, vastus lateralis, and gluteus maximus muscles at baseline (*p* > 0.05). At post-intervention, iEMG in the gastrocnemius, vastus medialis, and vastus lateralis muscles was significantly higher in the experimental group than in the control group (*p* < 0.05). Furthermore, within the experimental group, iEMG for all four muscles was significantly higher at post-test than pre-test (*p* < 0.001). Within the control group, iEMG for the gastrocnemius, vastus medialis, and vastus lateralis muscles was significantly higher at post-test than pre-test (*p* < 0.05). No significant differences in iEMG were observed across all ten muscles during the downward phase. Significant time × group interaction effects for iEMG were found in the tibialis anterior (*p* = 0.041), vastus medialis (*p* = 0.020), vastus lateralis (*p* = 0.018), and biceps femoris (*p* = 0.013) muscles during the downward phase. Simple effects analysis revealed no significant differences in iEMG between the experimental and control groups for any of the four muscles at baseline (*p* > 0.05). At post-test, the iEMG of the vastus medialis, vastus lateralis, and biceps femoris in the experimental group was significantly higher than that in the control group (*p* < 0.05), while the iEMG of the tibialis anterior was significantly lower than that in the control group (*p* < 0.05). Furthermore, within the experimental group, post-test iEMG of the vastus medialis, vastus lateralis, and biceps femoris was significantly higher than pre-test (*p* < 0.001), while iEMG of the tibialis anterior was significantly lower than pre-test (*p* < 0.001); Within the control group, posterior iEMG of the tibialis anterior was significantly lower than anterior iEMG (*p* < 0.05), while posterior iEMG of the vastus medialis and vastus lateralis was significantly higher than anterior iEMG (*p* < 0.05).

In the freestyle kick motion, as shown in Table 3, the interaction effect of time × group on RMS data for the gastrocnemius (*p* = 0.015), adductor magnus (*p* = 0.005), vastus lateralis (*p* = 0.001), and gluteus maximus (*p* = 0.024) muscles during the up-swing phase was significant. Simple effects analysis revealed no significant differences in RMS between the experimental and control groups for the gastrocnemius, vastus medialis, vastus lateralis, and gluteus maximus muscles at baseline (*p* > 0.05). At post-test, RMS values for all four muscles were significantly higher in the experimental group than in the control group (*p* < 0.05). Furthermore, within the experimental group, RMS values for all four muscles at post-test were significantly higher than at pre-test (*p* < 0.05). Within the control group, RMS values for the vastus medialis, vastus lateralis, and gluteus maximus at post-test were significantly higher than at pre-test (*p* < 0.05). No significant differences in RMS were observed for any muscle during the transition phase. In the downward phase, significant time × group interaction effects were observed for the RMS of the tibialis anterior (*p* = 0.032), vastus medialis (*p* = 0.027), and vastus lateralis (*p* = 0.030). Simple effects analysis revealed no significant differences in RMS between the experimental and control groups for these three muscles during the pre-test (*p* > 0.05). At post-test, the RMS of the tibialis anterior in the experimental group was significantly lower than that in the control group (*p* < 0.05). Furthermore, within the experimental group, the RMS of all three muscles at post-test differed significantly from their pre-test values (*p* < 0.05); within the control group, the RMS of the tibialis anterior was significantly lower than its pre-test value (*p* < 0.05).

### 3.2. Effects of NMES Strength Training on Muscle Synergy in the Kick Movement of Freestyle Swimmers

#### Muscle Synergy Patterns in Freestyle Kick Technique

**Figure 4:**
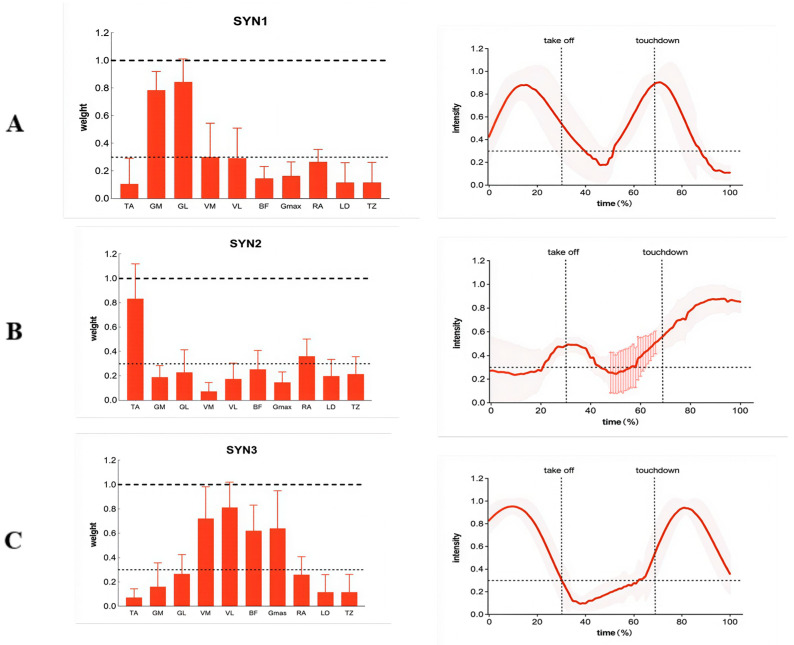
Muscle synergy patterns (top row) and their corresponding activation coefficients (bottom row) extracted from the freestyle kick. Data are presented as mean (solid line) ± standard deviation (shaded area). TA: Tibialis anterior; GM: Gastrocnemius medialis; GL: Gastrocnemius lateralis; VM: Vastus medialis; VL: Vastus lateralis; BF: Biceps femoris; Gmax: Gluteus maximus; RA: Rectus abdominis; LD: Latissimus dorsi; TZ: Trapezius. (**A**), Synergy 1; (**B**), Synergy 2; (**C**), Synergy 3.

In the freestyle kick motion, we performed cluster analysis on the electromyography data of the control group before intervention, forming three cluster centers as reference synergies. Figure 4A illustrates the muscle synergy patterns matched based on these three reference synergies under the pre-intervention conditions of the control group. We analyzed the functions of these three synergies by referencing previous literature and considering muscle activation weights and synergy activation coefficients within the synergies. Synergy I primarily reached activation peaks during the mid-Up-swing phase and early Downward phase, with significant activation weights in the medial and lateral gastrocnemius muscles and the biceps femoris. Synergy 2 primarily involves the tibialis anterior and rectus abdominis muscles, showing marked activation during the late Up-swing phase and most of the Downward phase. Synergy 3 mainly includes the vastus medialis, vastus lateralis, and gluteus maximus muscles, reaching peak activation during the early Up-swing phase and the middle Downward phase.

As shown in Table 4, NMES strength training did not produce a significant main effect or interaction effect on the average number of synergistic muscles activated during the freestyle kick.

### 3.3. Synergistic Structure

#### 3.3.1. Synergistic Structure in the Freestyle Kick

As shown in Table 5, in Synergy 1, the dominant muscles were the medial and lateral gastrocnemius muscles, with the medial and lateral vastus muscles also exhibiting some degree of activation. Comparisons using repeated measures ANOVA revealed no significant differences in the synergy weights of the ten muscles between the two groups before and after intervention. In Synergy 2, the primary activated muscles were the tibialis anterior and rectus abdominis, while other muscles showed lower activation weights. Repeated measures ANOVA revealed that within the experimental group, the tibialis anterior weight significantly decreased post-intervention (*p* = 0.030). Within the control group, the tibialis anterior weight showed a downward trend post-intervention but no significant difference between pre- and post-intervention. Additionally, group differences did not significantly alter muscle activation weights either pre- or post-intervention.

In the synergistic muscle group 3, the primary activated muscles were the vastus medialis, vastus lateralis, biceps femoris, and gluteus maximus. Repeated measures ANOVA revealed significant interaction effects for activation weights in the vastus medialis (*p* = 0.039) and biceps femoris (*p* = 0.035). Within the experimental group, activation weights for the vastus medialis and biceps femoris significantly increased post-intervention. Within the control group, no significant differences in activation weights for the vastus medialis and biceps femoris were observed before or after the intervention. Furthermore, group differences did not significantly alter muscle activation weights either before or after the intervention.

#### 3.3.2. Activation Coefficient in Freestyle Kick Movement

As shown in Table 6, the activation coefficient for Synergy 1 primarily exhibits a two-phase pattern, with pre-activation occurring during the Up-swing phase and prior to the Down-stroke. The activation peak ranges approximately between 59% and 71%. No significant main effects or interaction effects were observed for activation duration, peak timing, or onset timing between the two groups before and after intervention. For the activation coefficient of Synergy 2, minor activation occurred before and after the up-swing leg exits the water, with renewed activation before the down-swing and peak activation after the down-swing. Repeated measures ANOVA revealed a significant interaction effect for activation duration. Within the experimental group, activation duration significantly decreased post-intervention (*p* = 0.020); within the control group, no significant difference existed between pre- and post-intervention durations. Furthermore, no significant between-group differences in activation duration were observed either pre- or post-intervention.

For the activation coefficient of Synergy 3, activation commenced during the Up-swing phase, rapidly ascended to peak, then rapidly declined, followed by a pre-activation phase before ground contact. Peak activation timing showed no significant differences across the four scenarios, generally ranging from 9% to 15%. No significant main effects or interaction effects were observed for activation duration, peak timing, or onset timing before and after intervention in either group.

## 4. Discussion

This study investigated the effects of a 12-week intervention combining neuromuscular electrical stimulation (NMES) pretreatment with aquatic resistance training on the neuromuscular control of the freestyle kick in high-level swimmers. Compared to aquatic resistance training alone, the combined protocol led to two primary adaptations: (1) an increase in the activation magnitude of key lower-limb prime movers, and (2) a reorganization of muscle coordination patterns toward greater efficiency.

The observed increase in iEMG and RMS of the quadriceps (vastus medialis and lateralis) and hamstring (biceps femoris) muscles during both the upward and downward phases in the experimental group indicates enhanced neuromuscular recruitment. This is consistent with the known physiological effect of medium-frequency NMES (50 Hz), which effectively recruits high-threshold motor units and promotes synchronous firing [29]. The pre-activation of these muscle groups via NMES prior to water-based training likely facilitated a state of heightened neural excitability, allowing for greater motor unit mobilization during the subsequent sport-specific task [30]. This finding supports the use of NMES as a potent “neuromuscular primer” to augment the training stimulus of aquatic resistance exercises.

Beyond increased activation, the experimental group demonstrated a more refined muscle coordination strategy. The significant increase in the weighting of the vastus medialis and biceps femoris within the core propulsion synergy (SYN3) suggests that the nervous system not only recruited these muscles more forcefully but also assigned them a more dominant role within the motor program. Concurrently, the reduced activation duration of the postural adjustment synergy (SYN2) points to a faster, more transient engagement of muscles like the tibialis anterior and rectus abdominis. This streamlined activation pattern is characteristic of more skilled movement execution, where postural adjustments become quicker and less energy-consuming, freeing neural resources for the primary task of propulsion [31]. The shift toward a “proximal reinforcement, distal optimization” pattern—evidenced by increased proximal muscle activation alongside reduced tibialis anterior activity—aligns with the efficient, hip-driven kicking mechanics described in National Level swimmers [32]. The decreased distal activation may reflect a more relaxed ankle posture, potentially contributing to a more fluid “whip-like” motion and reduced drag [33].

The extraction of three consistent synergy modules aligns with the modular control theory for cyclic, multi-joint movements like swimming [34]. The fact that the number of synergies remained unchanged between groups suggests that the combined training did not alter the fundamental dimensionality of the control system but rather refined the internal structure and timing of existing modules. This indicates an optimization of pre-existing neural circuitry rather than the acquisition of a novel coordination pattern [35].

Limitations of the Study: This study is limited by the design of the post-intervention testing protocol, in which the experimental group performed a combined NMES + ART session immediately before testing, while the control group performed only an ART session. This discrepancy introduces a potential confounding effect, as the acute neuromuscular priming from NMES pretreatment in the experimental group may have influenced post-test muscle activation and synergy measures independently of the long-term training adaptations. Consequently, the observed differences between groups may partially reflect acute rather than chronic effects, limiting the ability to isolate the specific contribution of the 12-week integrated training intervention. Future studies should standardize pre-test conditions or include a washout period to ensure that post-intervention assessments purely reflect cumulative training adaptations.

## 5. Conclusions

This 12-week randomized controlled trial demonstrated that combining NMES pretreatment with aquatic resistance training induces distinct neuromuscular adaptations in the freestyle kick of National Level swimmers, compared to aquatic resistance training alone. The combined protocol led to enhanced activation of key lower-limb propulsive muscles and optimized the organization of muscle synergy patterns, specifically through increased weighting of prime movers within the core propulsion module (SYN3) and reduced activation duration of the postural adjustment module (SYN2). These findings indicate that the intervention not only augmented peripheral muscle recruitment capacity but also refined the central nervous system’s efficiency in coordinating muscle resources. Therefore, integrating NMES as a preparatory stimulus with sport-specific aquatic training presents a viable strategy for targeted neuromuscular enhancement in swimming, offering a novel theoretical and methodological framework for advanced training design.

## Figures and Tables

**Table 1:** Summary of the 12-week training interventions for the control (CON) and experimental (EXP) groups.

Component	Control Group (CON)	Experimental Group (EXP)
1. Intervention Core	Aquatic Resistance Training (ART) only.	NMES Pretreatment + Aquatic Resistance Training (ART).
2. NMES Pretreatment	Not applicable.	Protocol: Applied on land immediately before ART. Position: Supine. Target Muscles: Vastus medialis, vastus lateralis, biceps femoris. Parameters: 50 Hz frequency, 200 µs pulse width, biphasic symmetric rectangular waveform. Duty Cycle: 10 s ON/30 s OFF. Duration: 3 min total. Intensity: 80–90% of maximum tolerated contraction (visible, strong contraction without discomfort). Transition to Water: Within 5 min post-stimulation.
3. Aquatic Resistance Training (ART)	Content: 25 m maximal freestyle kicking sprints with short-bladed fins. Technique Emphasis: Hip-driven “whip kick”. Intensity: 75–80% of individual maximum kicking speed. Volume: 4 sets × 25 m. Rest Interval: 2.5 min between sets. Progression: Load adjusted every two weeks based on performance.	Content: 25-m maximal freestyle kicking sprints with short-bladed fins. Technique Emphasis: Hip-driven “whip kick”. Intensity: 75–80% of individual maximum kicking speed. Volume: 4 sets × 25 m. Rest Interval: 2.5 min between sets. Progression: Load adjusted every two weeks based on performance.
4. Post-Intervention Testing	Testing (i.e., the single post-intervention assessment) commenced within 8 min after completing a standard ART session.	Testing (i.e., the single post-intervention assessment) commenced within 8 min after completing a combined NMES + ART session.

**Table 2:** Effects of NMES Strength Training on iEMG During Freestyle Kick (M ± SD, μV·s).

Phase	Muscle	Experimental Group		Control Group		Time × Group Interaction (*p*)	ηp^2^
		Pre-Intervention	Post-Intervention	Pre-Intervention	Post-Intervention		
Up-swing phase	Tibialis anterior	23.64 ± 4.17	21.90 ± 9.78	22.26 ± 3.38	21.63 ± 7.19	0.851	0.002
	Gastrocnemius medialis	70.64 ± 15.27	73.22 ± 15.05	69.48 ± 16.81	72.15 ± 13.58	0.943	<0.001
	Gastrocnemius lateralis	69.52 ± 12.15	82.04 ± 14.65 *	67.49 ± 14.37	75.84 ± 13.62	0.010	0.42
	Adductor longus	69.73 ± 11.66	86.12 ± 14.98 *	70.78 ± 13.70	79.02 ± 13.57	0.021	0.48
	Adductor magnus	66.54 ± 13.45	88.31 ± 14.09 *	67.96 ± 13.93	77.69 ± 11.99	0.011	0.41
	Biceps femoris	38.32 ± 6.85	40.72 ± 7.16	36.39 ± 9.97	38.00 ± 7.63	0.642	0.008
	Gluteus maximus	60.08 ± 7.46	72.85 ± 12.10 *	62.28 ± 8.59	67.33 ± 10.62	0.035	0.35
	Rectus abdominis	35.74 ± 5.90	34.88 ± 7.62	33.37 ± 7.97	34.90 ± 8.21	0.430	0.024
	latissimus dorsi	26.33 ± 7.62	23.19 ± 10.65	27.12 ± 6.59	28.55 ± 10.70	0.185	0.062
	trapezius	22.87 ± 9.29	25.64 ± 11.88	21.51 ± 7.38	20.71 ± 11.02	0.317	0.036
Downward phase	Tibialis anterior	68.50 ± 13.17	56.19 ± 12.07 *	70.37 ± 15.54	62.72 ± 13.91	0.041	0.34
	Gastrocnemius medialis	59.64 ± 8.27	66.27 ± 15.80	57.90 ± 9.73	61.10 ± 8.71	0.478	0.020
	Gastrocnemius lateralis	60.52 ± 9.15	67.52 ± 11.83	59.73 ± 10.06	64.16 ± 12.47	0.600	0.010
	Adductor longus	55.23 ± 8.71	68.71 ± 9.83 *	56.85 ± 8.50	62.09 ± 10.22	0.020	0.39
	Adductor magnus	54.58 ± 9.60	69.77 ± 11.80 *	55.21 ± 10.12	62.91 ± 10.87	0.018	0.40
	Biceps femoris	39.76 ± 7.65	48.69 ± 10.83 *	36.84 ± 8.29	40.72 ± 9.12	0.013	0.43
	Gluteus maximus	50.64 ± 9.02	54.90 ± 10.81	52.42 ± 10.17	55.77 ± 11.20	0.837	0.003
	Rectus abdominis	38.47 ± 6.74	37.12 ± 10.39	40.12 ± 9.56	36.98 ± 9.71	0.562	0.012
	latissimus dorsi	26.80 ± 7.91	27.70 ± 9.47	25.73 ± 6.08	26.88 ± 8.02	0.906	<0.001
	trapezius	24.04 ± 6.36	25.98 ± 5.28	25.16 ± 8.18	25.70 ± 7.01	0.402	0.027
Transition phase	Tibialis anterior	38.42 ± 9.02	33.11 ± 8.57	35.74 ± 10.11	34.20 ± 9.08	0.252	0.045
	Gastrocnemius medialis	20.52 ± 9.50	19.76 ± 10.26	22.80 ± 9.43	25.86 ± 10.36	0.127	0.081
	Gastrocnemius lateralis	19.53 ± 8.43	20.63 ± 8.96	19.72 ± 9.57	19.36 ± 10.48	0.694	0.006
	Adductor longus	20.48 ± 9.92	24.67 ± 8.92	19.37 ± 10.10	20.56 ± 9.88	0.210	0.054
	Adductor magnus	21.48 ± 10.49	17.59 ± 11.69	20.77 ± 9.04	19.50 ± 9.63	0.179	0.064
	Biceps femoris	29.53 ± 6.02	24.72 ± 10.59	27.69 ± 7.36	28.63 ± 8.79	0.095	0.098
	Gluteus maximus	20.92 ± 9.39	24.72 ± 10.73	21.73 ± 8.50	22.63 ± 9.61	0.581	0.011
	Rectus abdominis	46.35 ± 11.70	44.80 ± 12.63	47.18 ± 12.34	46.01 ± 11.26	0.844	0.002
	latissimus dorsi	33.33 ± 10.74	34.72 ± 12.05	34.84 ± 10.17	33.92 ± 11.90	0.376	0.030
	trapezius	29.38 ± 10.80	30.00 ± 11.09	26.57 ± 12.29	28.79 ± 10.92	0.733	0.005

Note: *: Time × Group interaction effect, *p* < 0.05. The experimental group underwent NMES combined with aquatic resistance training. The control group underwent traditional aquatic resistance training.

**Table 3:** Effects of NMES Strength Training on RMS in Freestyle Kick Movement (M ± SD, μV).

Phase	Muscle	Experimental Group		Control Group		Time × Group Interaction (*p*)	ηp^2^
		Pre-Intervention	Post-Intervention	Pre-Intervention	Post-Intervention		
Up-swing phase	Tibialis anterior	32.10 ± 10.73	28.07 ± 10.17	30.75 ± 12.39	27.89 ± 13.61	0.891	0.001
	Gastrocnemius medialis	110.45 ± 20.35	118.57 ± 23.48	114.40 ± 22.04	119.83 ± 23.86	0.722	0.005
	Gastrocnemius lateralis	109.02 ± 21.13	119.29 ± 22.41 *	105.59 ± 25.90	110.82 ± 19.30	0.015	0.48
	Adductor longus	97.38 ± 25.56	121.01 ± 29.89 *	99.93 ± 24.97	109.81 ± 23.62	0.005	0.54
	Adductor magnus	99.42 ± 23.90	129.75 ± 29.00 *	98.13 ± 27.88	112.75 ± 30.89	0.001	0.69
	Biceps femoris	51.91 ± 11.27	59.92 ± 13.41	48.48 ± 15.58	53.10 ± 14.09	0.325	0.035
	Gluteus maximus	88.36 ± 19.57	111.38 ± 21.70 *	90.84 ± 20.75	99.16 ± 19.83	0.024	0.36
	Rectus abdominis	44.23 ± 10.11	41.80 ± 11.35	45.83 ± 12.38	44.73 ± 13.90	0.612	0.010
	latissimus dorsi	31.84 ± 12.70	35.69 ± 18.09	28.86 ± 10.75	27.95 ± 12.77	0.155	0.068
	trapezius	30.20 ± 9.15	31.67 ± 10.63	31.27 ± 8.53	31.99 ± 10.51	0.834	0.003
Downward phase	Tibialis anterior	99.16 ± 16.82	84.15 ± 17.22 *	101.64 ± 19.67	90.38 ± 18.92	0.032	0.35
	Gastrocnemius medialis	80.57 ± 19.73	85.16 ± 17.83	78.13 ± 18.29	81.18 ± 17.64	0.865	0.002
	Gastrocnemius lateralis	81.09 ± 16.86	85.69 ± 18.33	82.75 ± 19.63	84.87 ± 20.93	0.598	0.011
	Adductor longus	78.47 ± 16.27	88.15 ± 18.90 *	80.86 ± 18.75	85.79 ± 17.45	0.027	0.46
	Adductor magnus	76.33 ± 17.53	84.94 ± 19.12 *	78.21 ± 20.11	83.50 ± 19.37	0.030	0.35
	Biceps femoris	58.46 ± 6.39	61.77 ± 10.31	57.01 ± 12.53	58.13 ± 14.83	0.478	0.020
	Gluteus maximus	70.64 ± 19.02	77.90 ± 15.62	72.74 ± 21.39	76.66 ± 18.10	0.554	0.013
	Rectus abdominis	55.88 ± 7.62	56.92 ± 10.42	57.09 ± 16.31	55.28 ± 13.52	0.381	0.029
	latissimus dorsi	44.71 ± 7.50	41.58 ± 10.90	42.56 ± 10.54	40.92 ± 13.82	0.911	<0.001
	trapezius	43.19 ± 6.67	43.52 ± 7.81	44.21 ± 10.74	40.85 ± 9.51	0.105	0.092
Transition phase	Tibialis anterior	50.35 ± 10.69	51.29 ± 13.07	52.85 ± 12.36	51.05 ± 11.95	0.442	0.023
	Gastrocnemius medialis	31.21 ± 8.26	33.10 ± 7.69	33.75 ± 10.15	33.86 ± 10.88	0.678	0.007
	Gastrocnemius lateralis	32.47 ± 8.93	33.85 ± 9.79	30.31 ± 9.25	33.67 ± 8.52	0.380	0.029
	Adductor longus	29.77 ± 6.58	27.65 ± 9.06	31.80 ± 7.36	32.80 ± 10.11	0.410	0.026
	Adductor magnus	30.22 ± 10.78	33.91 ± 9.84	29.74 ± 9.58	30.29 ± 9.31	0.295	0.038
	Biceps femoris	35.39 ± 8.48	31.58 ± 10.52	33.62 ± 9.03	35.86 ± 10.81	0.052	0.120
	Gluteus maximus	29.65 ± 10.21	32.52 ± 9.53	30.93 ± 11.86	33.62 ± 14.72	0.936	<0.001
	Rectus abdominis	63.78 ± 19.26	67.82 ± 21.52	65.64 ± 18.96	66.92 ± 19.93	0.715	0.005
	latissimus dorsi	40.49 ± 11.28	42.82 ± 13.84	38.63 ± 10.46	37.90 ± 13.63	0.270	0.042
	trapezius	37.13 ± 11.70	38.82 ± 14.85	38.72 ± 10.29	36.02 ± 15.87	0.185	0.062

Note: *: Time × Group interaction effect, *p* < 0.05.

**Table 4:** Average Number of Coordinated Movements per Swimmer and Frequency of Each Coordinated Movement in Freestyle Kick Technique.

Synergy	Experimental Group		Control Group	
	Pre-Intervention	Post-Intervention	Pre-Intervention	Post-Intervention
N_SYN_	2.90 ± 0.57	2.90 ± 0.32	3.10 ± 0.57	3.00 ± 0.47
N_SYN1_	27	27	29	25
N_SYN2_	20	20	20	22
N_SYN3_	18	20	18	20
N_U-SYN_	7	5	10	8

Note: NSYN: Number of synergies per person; NSYN1…3: Number of occurrences of synergies SYN1–3; NU-SYN: Number of unmatched synergies.

**Table 5:** Muscle Synergy Patterns for SYN1 in Freestyle Kick Movement.

Synergy	Muscle	Experimental Group		Control Group		Time × Group Interaction (*p*)	ηp^2^
		Pre-Intervention	Post-Intervention	Pre-Intervention	Post-Intervention		
SYN1	Tibialis anterior	0.09 ± 0.07	0.12 ± 0.05	0.11 ± 0.12	0.13 ± 0.08	0.752	0.004
	Gastrocnemius medialis	0.82 ± 0.19	0.90 ± 0.24	0.79 ± 0.10	0.82 ± 0.17	0.685	0.006
	Gastrocnemius lateralis	0.79 ± 0.15	0.84 ± 0.21	0.85 ± 0.20	0.88 ± 0.19	0.812	0.002
	Adductor longus	0.31 ± 0.12	0.38 ± 0.16	0.30 ± 0.26	0.29 ± 0.15	0.124	0.082
	Adductor magnus	0.31 ± 0.14	0.37 ± 0.18	0.29 ± 0.23	0.32 ± 0.14	0.332	0.034
	Biceps femoris	0.10 ± 0.02	0.15 ± 0.10	0.15 ± 0.09	0.13 ± 0.11	0.089	0.101
	Gluteus maximus	0.11 ± 0.06	0.13 ± 0.07	0.16 ± 0.13	0.17 ± 0.11	0.955	<0.001
	Rectus abdominis	0.30 ± 0.13	0.29 ± 0.11	0.27 ± 0.09	0.30 ± 0.16	0.421	0.025
	latissimus dorsi	0.10 ± 0.08	0.12 ± 0.11	0.12 ± 0.11	0.11 ± 0.08	0.667	0.007
	trapezius	0.14 ± 0.09	0.13 ± 0.06	0.12 ± 0.12	0.12 ± 0.09	0.591	0.011
SYN2	Tibialis anterior	0.80 ± 0.21	0.39 ± 0.13 *	0.83 ± 0.29	0.77 ± 0.24	0.030	0.45
	Gastrocnemius medialis	0.24 ± 0.14	0.28 ± 0.10	0.19 ± 0.11	0.20 ± 0.07	0.478	0.020
	Gastrocnemius lateralis	0.26 ± 0.17	0.19 ± 0.07	0.23 ± 0.19	0.25 ± 0.12	0.103	0.094
	Adductor longus	0.11 ± 0.08	0.14 ± 0.11	0.07 ± 0.05	0.15 ± 0.10	0.401	0.027
	Adductor magnus	0.20 ± 0.11	0.25 ± 0.15	0.17 ± 0.10	0.22 ± 0.03	0.881	0.001
	Biceps femoris	0.21 ± 0.12	0.17 ± 0.06	0.25 ± 0.11	0.18 ± 0.16	0.733	0.005
	Gluteus maximus	0.18 ± 0.14	0.13 ± 0.04	0.15 ± 0.19	0.19 ± 0.13	0.053	0.118
	Rectus abdominis	0.40 ± 0.21	0.47 ± 0.13	0.36 ± 0.19	0.34 ± 0.16	0.105	0.092
	latissimus dorsi	0.15 ± 0.09	0.11 ± 0.04	0.20 ± 0.13	0.22 ± 0.02	0.176	0.063
	trapezius	0.17 ± 0.13	0.13 ± 0.06	0.22 ± 0.14	0.25 ± 0.11	0.316	0.036
SYN3	Tibialis anterior	0.13 ± 0.08	0.20 ± 0.06	0.07 ± 0.04	0.11 ± 0.07	0.642	0.008
	Gastrocnemius medialis	0.25 ± 0.14	0.31 ± 0.12	0.16 ± 0.19	0.20 ± 0.12	0.724	0.005
	Gastrocnemius lateralis	0.24 ± 0.17	0.22 ± 0.15	0.27 ± 0.15	0.25 ± 0.08	0.501	0.017
	Adductor longus	0.68 ± 0.20	0.97 ± 0.09 *	0.72 ± 0.29	0.80 ± 0.20	0.039	0.34
	Adductor magnus	0.87 ± 0.13	0.91 ± 0.15	0.81 ± 0.23	0.89 ± 0.19	0.502	0.017
	Biceps femoris	0.56 ± 0.21	0.87 ± 0.14 *	0.62 ± 0.24	0.67 ± 0.16	0.035	0.35
	Gluteus maximus	0.70 ± 0.21	0.75 ± 0.15	0.64 ± 0.31	0.70 ± 0.13	0.948	<0.001
	Rectus abdominis	0.20 ± 0.16	0.24 ± 0.07	0.26 ± 0.15	0.24 ± 0.06	0.224	0.051
	latissimus dorsi	0.17 ± 0.04	0.18 ± 0.07	0.12 ± 0.16	0.15 ± 0.10	0.882	0.001
	trapezius	0.08 ± 0.01	0.13 ± 0.08	0.12 ± 0.16	0.17 ± 0.08	0.742	0.004

Note: *: Time × Group interaction effect, *p* < 0.05.

**Table 6:** Activation Coefficients of SYN in the Synergistic Pattern During Freestyle Kick Movement.

**Activation Coefficient**	**Parameters**	**Experimental Group**		**Control Group**		**Time × Group Interaction (*p*)**	**ηp^2^**
		**Pre-Intervention**	**Post-Intervention**	**Pre-Intervention**	**Post-Intervention**		
SYN1	Activation Duration T	0.79 ± 0.19	0.85 ± 0.13	0.76 ± 0.28	0.83 ± 0.20	0.915	<0.001
	Peak Moment Tmax	0.68 ± 0.24	0.60 ± 0.16	0.71 ± 0.21	0.59 ± 0.25	0.702	0.006
	The Moment Begins Tstart	0.27 ± 0.16	0.25 ± 0.08	0.24 ± 0.11	0.18 ± 0.10	0.442	0.023
SYN2	Activation Duration T	0.69 ± 0.23	0.37 ± 0.14 *	0.65 ± 0.26	0.57 ± 0.18	0.020	0.39
	Peak Moment Tmax	0.90 ± 0.26	0.87 ± 0.18	0.93 ± 0.30	0.88 ± 0.23	0.721	0.005
	The Moment Begins Tstart	0.17 ± 0.07	0.27 ± 0.05	0.20 ± 0.09	0.24 ± 0.11	0.064	0.114
SYN3	Activation Duration T	0.69 ± 0.18	0.63 ± 0.14	0.68 ± 0.20	0.72 ± 0.16	0.051	0.120
	Peak Moment Tmax	0.09 ± 0.04	0.14 ± 0.08	0.10 ± 0.05	0.15 ± 0.09	0.941	<0.001
	The Moment Begins Tstart	0.64 ± 0.27	0.67 ± 0.26	0.62 ± 0.26	0.57 ± 0.24	0.259	0.043

Note: *: Time × Group interaction effect, *p* < 0.05.

## Data Availability

The original contributions presented in this study are included in the article. Further inquiries can be directed to the corresponding author.
